# Local stability properties of complex, species‐rich soil food webs with functional block structure

**DOI:** 10.1002/ece3.8278

**Published:** 2021-11-03

**Authors:** Francisco de Castro, Sina M. Adl, Stefano Allesina, Richard D. Bardgett, Thomas Bolger, Johnathan J. Dalzell, Mark Emmerson, Thomas Fleming, Diego Garlaschelli, Jacopo Grilli, Silja Emilia Hannula, Franciska de Vries, Zoë Lindo, Aaron G. Maule, Maarja Öpik, Matthias C. Rillig, Stavros D. Veresoglou, Diana H. Wall, Tancredi Caruso

**Affiliations:** ^1^ Fisheries & Aquatic Ecosystems Agri‐Food & Biosciences Institute Belfast UK; ^2^ Department of Soil Science University of Saskatchewan Saskatoon SK Canada; ^3^ Department of Ecology & Evolution and Computation Institute University of Chicago Chicago Illinois USA; ^4^ Department of Earth and Environmental Sciences The University of Manchester Manchester UK; ^5^ School of Biology & Environmental Science University College Dublin Dublin 4 Ireland; ^6^ Grassland & Plant Science Agri‐Food & Biosciences Institute Belfast UK; ^7^ School of Biological Sciences and Institute for Global Food Security Queen's University of Belfast Belfast UK; ^8^ IMT School of Advanced Studies Lucca Italy; ^9^ Instituut‐Lorentz for Theoretical Physics Leiden Institute of Physics University of Leiden Leiden The Netherlands; ^10^ The Abdus Salam International Centre for Theoretical Physics Quantitative Life Science Section Trieste Italy; ^11^ Department of Terrestrial Ecology Netherlands Institute of Ecology (NIOO‐KNAW) Wageningen The Netherlands; ^12^ Institute for Biodiversity and Ecosystem Dynamics University of Amsterdam Amsterdam The Netherlands; ^13^ Department of Biology The University of Western Ontario London ON Canada; ^14^ Department of Botany University of Tartu Tartu Estonia; ^15^ Institut für Biologie Freie Universität Berlin Berlin Germany; ^16^ Department of Biology School of Global Environmental Sustainability Colorado State University Fort Collins Colorado USA

**Keywords:** block structure, complexity, food webs, functional structure, soil, species, stability

## Abstract

Ecologists have long debated the properties that confer stability to complex, species‐rich ecological networks. Species‐level soil food webs are large and structured networks of central importance to ecosystem functioning. Here, we conducted an analysis of the stability properties of an up‐to‐date set of theoretical soil food web models that account both for realistic levels of species richness and the most recent views on the topological structure (who is connected to whom) of these food webs. The stability of the network was best explained by two factors: strong correlations between interaction strengths and the blocked, nonrandom trophic structure of the web. These two factors could stabilize our model food webs even at the high levels of species richness that are typically found in soil, and that would make random systems very unstable. Also, the stability of our soil food webs is well‐approximated by the cascade model. This result suggests that stability could emerge from the hierarchical structure of the functional organization of the web. Our study shows that under the assumption of equilibrium and small perturbations, theoretical soil food webs possess a topological structure that allows them to be complex yet more locally stable than their random counterpart. In particular, results strongly support the general hypothesis that the stability of rich and complex soil food webs is mostly driven by correlations in interaction strength and the organization of the soil food web into functional groups. The implication is that in real‐world food web, any force disrupting the functional structure and distribution pattern of interaction strengths (i.e., energy fluxes) of the soil food webs will destabilize the dynamics of the system, leading to species extinction and major changes in the relative abundances of species.

## Graphical abstract

Species‐level soil food webs are very large and structured networks. We analysed the stability properties of these networks. Correlation in interaction strength and organization by functional blocks stabilised soil food webs.

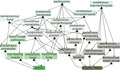

## 1 INTRODUCTION

The study of the stability of ecological communities has a long history (Allesina et al., 2015; Goodman, 1975; Grilli et al., 2017; Jacquet et al., 2016; May, 1972, 1973; Moore & Hunt, 1988; Rooney et al., 2006) but is a much debated topic (Donohue et al., 2013; Grimm & Wissel, 1997; Loreau & de Mazancourt, 2013). Much of this debate is focused on the relationship between stability and complexity: Ecological networks appear extremely complex in terms of species richness and connections, which are central to the topology of the web (i.e., whom is connected to whom), and interaction strengths, which control energy fluxes between species. Yet natural communities are also relatively stable over a broad range of spatial and temporal scales (Paine, 1992; Pimm, 2002). In contrast, complex but randomly assembled mathematical models of ecological networks are dynamically unstable, even when subjected to small perturbations (Allesina & Tang, 2012; May, 1973). The structure of real ecological networks is not random, however, and ecologists investigate what characteristics generate stability in real ecological networks both empirically and theoretically (Donohue et al., 2013; Grilli et al., 2017; O'Gorman & Emmerson, 2009). Possibly, the first and simplest example of a nonrandom food web model was the cascade model, in which species are ordered along a monodimensional hierarchy and each species feeds randomly only on species that have a lower rank in this hierarchy (Cohen et al., 2012; Cohen & Newman, 1985). More models have then been developed with increasing levels of complexity and realism (Allesina et al., 2008).

In this context, soil food webs have attracted much attention, especially with regard to our understanding of the properties and mechanisms that give rise to stability (de Ruiter et al., 1995; de Vries et al., 2012; McCann et al., 2005; Moore & Hunt, 1988; Moore & de Ruiter, 2012; Rooney et al., 2006). Soil food webs are particularly interesting because they are both exceptionally diverse at the species and functional levels and have complex patterns of interconnections, which implies a clearly nonrandom structure in the topology of the food web. Also, linkages between soil communities and aboveground biota play a pivotal role in regulating ecosystem processes, such as nitrogen and carbon cycling (Adl et al., 2020; Bardgett et al., 2008; De Deyn et al., 2008; de Vries et al., 2012; Wardle et al., 2004).

Some structural properties of soil food webs are considered to be of particular importance for their stability. For example, species at the higher trophic levels may connect the distinct energy channels (e.g., fungal or bacterial channel, see below) that are the major pathways for energy flow in soil food webs (Moore & de Ruiter, 2012). Theoretically, the coupling of these energy channels can stabilize the dynamics of soil food webs (Rooney & McCann, 2012; Rooney et al., 2006).

**FIGURE 1:**
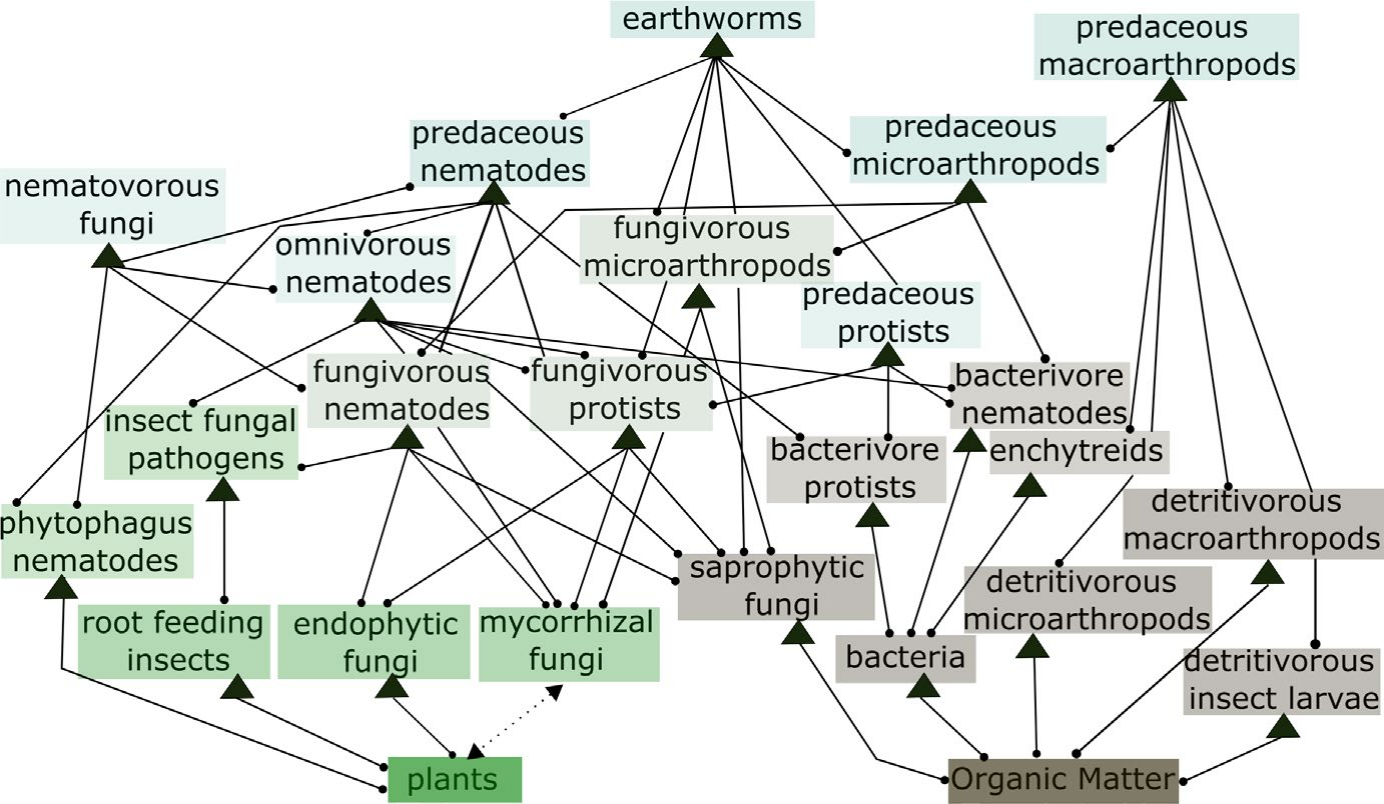
Topology of the analyzed soil food web model (see also Table S1 and references therein). We include more functional groups and interactions than in the classical soil food web models. These interactions are now considered to play a key role, and some of the links in our food web have been underestimated in the past. One major effect of this increased complexity is that the distinction between the traditional energy channels blurs. Examples of these groups and interactions are the protists and their interactions with both microbes and nematodes. The model also includes groups that interact strongly with plant roots and that can be resources to soil fauna such as phytophagous nematodes or fungal pathogen of insects, which may control root‐feeding insects. Or also the group of endophytic fungi, different from mycorrhizal fungi, may be influenced positively by plants but may have subtly negative or neutral effects on their plant host (see Table S1 for full references). Some of the interactions are not drawn to facilitate the visualization of the web (see Table S1 for the full network matrix and Figure 2 for a species‐level stochastic realization of this network of interactions)

There are many aspects of the structure of soil food webs that are yet to be investigated as factors of stability. One is that soil networks are phenomenally species‐rich (Bardgett & van der Putten, 2014; Mora et al., 2011), but theoretical and empirical analyses of their stability has been based on aggregated data at the trophic group level, thereby neglecting the enormous diversity observed within trophic groups and its consequences on the network dynamics. A second point is that views on the functional structure (i.e., who is interacting with whom) of soil food webs is changing based on growing empirical data on several trophic and functional interactions that have been overlooked or underestimated in the past (Bradford, 2016). For example, fungal and bacterial energy channels are much more interconnected than previously thought (Ballhausen & de Boer, 2016; de Vries & Caruso, 2016; Wolkovich, 2016), due to the diversity and functional versatility of key groups, such as protists, which has been underestimated (Adl & Gupta, 2006; Averill, 2016; Geisen, 2016; Soong & Nielsen, 2016). Soil food webs are also highly size‐structured because they consist of taxa that differ in size, for example, nematodes, microarthropods, and earthworms, and that at the same time can be classified in well‐defined functional groups such as predaceous mites or bacterial feeding nematodes (Coleman et al., 2004). This organization of the soil food web potentially creates a hierarchy (Figure 1), which makes the soil food web model partially analogous to a simple cascade model (Cohen et al., 2012; Cohen & Newman, 1985). Another structural but quantitative aspect that could stabilize the soil food web is strong correlation of predator–prey interaction strengths (Tang et al., 2014). These correlations imply that for example, a strong effect of a consumer on their resource may generate a proportionally strong or weak effect of the resource on the consumer. In the lack of this correlation, the strength of the effect of the resource on the consumer would not predict the strength of the consumer on the resource, or vice versa. Negative correlations of predator–prey interaction strengths stabilize both structured and randomly constructed food webs (Tang et al., 2014). We, thus, hypothesize that correlations of reciprocal effects of predator–prey pairs can stabilize the dynamics of topologies such as those displayed by soil food webs (Tang et al., 2014).

Here, we integrate recent biological knowledge of the soil food web with a classical (i.e., random matrix theory) analysis of local stability in food webs (May, 1972; Tang et al., 2014) to investigate the local stability properties of a general soil food web model. We constructed theoretical soil food webs with an emphasis on their topology using realistic levels of species richness (i.e., node number) and feeding connections (whom is connected to whom). In practice, we used our collective knowledge of the literature to create a plausible and general topology (number of species and who is connected to whom) of the soil food webs. We then used random matrix theory (May, 1972; Tang et al., 2014) to generate the weights of the links and evaluate the implication of the constructed topology for the local stability properties of the population dynamics of these species‐rich soil food web models. We asked the following questions: (1) How is the stability of model soil food webs with a realistic topology (who is connected to whom) affected by plausible levels of species richness? (2) Given this topology, how does interaction strength, its variance, and the correlation between the effects of predators on prey, and vice versa, affect stability? (3) How does the cascade model, which nowadays represents a simple topological and almost null model, approximate the stability of large soil food webs? (4) Whether the constructed soil food webs are more stable than random predator–prey webs of comparable size and complexity?

We tested these hypotheses with the overarching goal of assessing how plausible and generally large levels of species richness as well as the recent view of the topological properties of soil food webs impact the dynamic stability of belowground ecological networks, which are of vital importance for ecosystem functioning and represent an ideal model for large (i.e., very many species) and highly structured (i.e., nonrandom topologies) networks (Allesina et al., 2015; Allesina & Tang, 2012; Grilli et al., 2017).

## 2 METHODS

### 2.1 Model definition (topology and real world information in the model)

Classic ecological work on theoretical food webs and local dynamic stability is based on the random matrix theory, but real food webs have a nonrandom topological and quantitative structure. We, thus, started from the classic soil food web model of Hunt (1987) to formulate a plausible topology of soil food web models and then compare the effect of this topology with those of random topologies. In this sense, the real‐world information we used in our models as parameters are the number of nodes in the web, the connectance of the web, and the overarching topology of the web. Hunt's model has been the basis of many studies of soil food webs (de Ruiter et al., 1995; de Vries et al., 2012; Rooney et al., 2006) and is based on the idea that soil food webs show three major energy channels: (i) the “brown” fungal channel based on slow processing of detritus; (ii) the “brown” bacterial channel based on fast processing of detritus; and (iii) the “green” root channel, which stems directly from plant roots and the mycorrhizal fungi. At the topological level, in the classic soil food web model, there is a relatively clear separation of lower trophic levels into fungal vs. bacterial feeders, although this separation blurs at higher trophic positions. However, given the high degree of omnivory and trophic/functional flexibility of many soil organisms (Adl & Gupta, 2006; Ballhausen & de Boer, 2016; de Vries & Caruso, 2016; Wolkovich, 2016), it is likely that even lower trophic‐level consumers obtain energy from all channels; this is certainly the case for the most abundant and diverse groups, namely, protists and nematodes, and possibly for a number of microarthropods (Ballhausen & de Boer, 2016; de Vries & Caruso, 2016; Wolkovich, 2016). The structure of our soil food web model, thus, included the classical channels but embedded them in the complexity that we now know characterize soil food webs and which blur the distinction between energy channels into a continuum (de Vries & Caruso, 2016). To the best of our knowledge, there are not empirical data that allow a full parameterization of a real soil food web while reflecting all the interactions taking place in soil food webs at the species level and also the fact that traditional energy channels are much more connected than believed in the past (see the role of protists, nematodes, and, more generally, omnivorous species). To overcome this issue, we, thus, made a number of conservative assumptions to limit ourselves to construct theoretical models that are plausible in terms of functional topology, that is, the connection between the major functional and trophic groups in the food web. Our “parameters” taken from the real world were, thus, topological. We expanded the classic food web model and represented this expanded model by a diet matrix of 24 functional groups (Figure 1 and Table S1 and “Groups_Interactions_REV.csv” in Appendix S1) that includes what, in our view, is the most up‐to‐date information on the structure of a general soil food web. We mostly based the model on temperate grassland food webs, but the model is not intended to describe any specific soil community or habitat, but rather a standard food web that represents the main characteristics of soil communities in general.

### 2.2 Evaluation of stability – Step 1: link weights and node numbers (richness)

**FIGURE 2:**
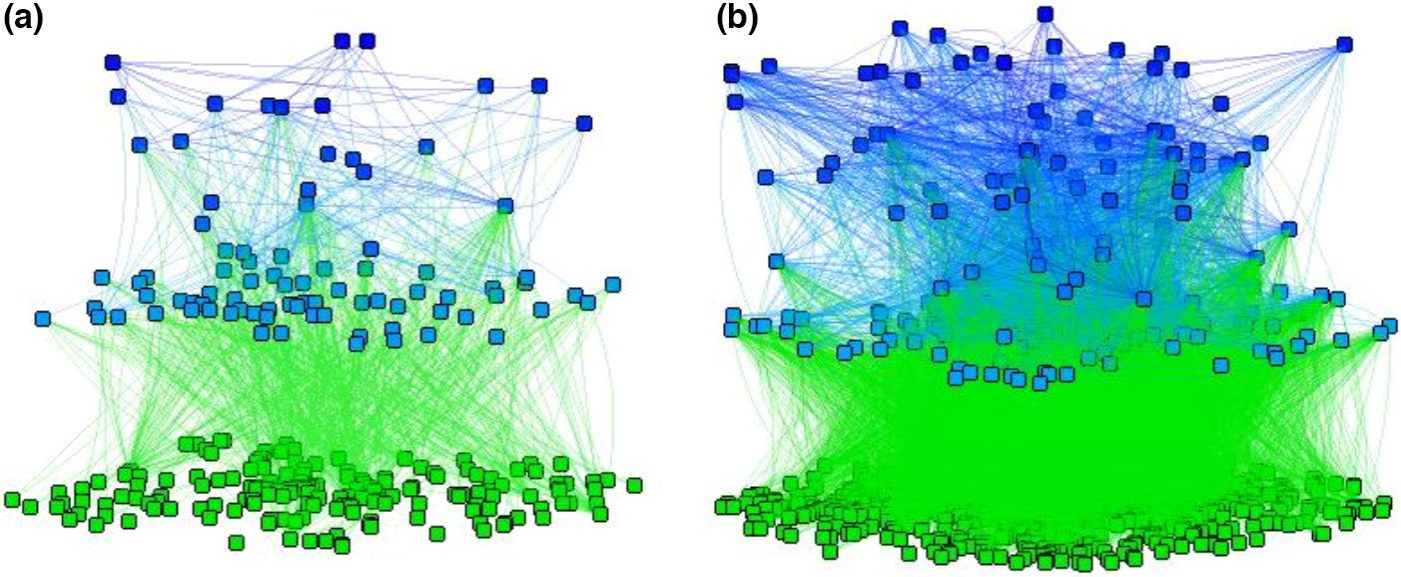
General examples of the species‐level soil food webs analyzed in this work. The food webs are built starting from the overarching functional topology, as shown in Figure 1. In real food webs, each of the groups of organisms in Figure 1 is actually represented by a variable number of species with interactions between species arranged by functional groups. (a) A “small” food web obtained by keeping the species richness of each group at the lowest level (see Table S2. (b) A “large” food web obtained by setting species richness of each group at its maximum (Table S2). The vertical axis and colors show species trophic level: in soil food webs, trophic levels are not discrete and the traditional distinction between energy channels is blurred by the intermediate positions of species that have a range of resources. Greener species are at the bottom of the web (plants, bacteria, saprotrophic fungi, and animals obtaining their energy directly or mostly from detritus). Shades of blue indicate the trophic level of higher level consumers and predators, with top predators in dark blue

Once we defined the web topology, we evaluated the stability of the systems following various steps. First, to incorporate into the model plausible levels of species richness, we generated multiple species‐level replicates of the functional group food web. We assumed that most local soil food webs involve the 24 groups identified in the previous step (Figure 1), although each group is represented by a different number of species at each specific location, and actual interaction, thus, takes place at the species level. To specify species richness within groups, we used species richness range from the literature (at the scale of 1 hectare at any sampling point in time: see Tables S2 and S3 and references therein for details). The species‐level replicates of the general model ranged from 500 to 3000 species (Figure 2). The range of species richness used to implement our simulations is meant to represent the order of magnitude typical of field surveys (see Supporting Information and “Groups_Richness_rev.csv” in Appendix S1) and is not meant to be accurate estimates at the chosen spatial scale (1 ha), rather they mostly reflect the fact that microbial diversity (bacteria, archaea, fungi, and protists) is much higher than animal diversity and that different groups of animals differ in species richness.

As in most analysis of food web dynamic stability, we assumed that a local equilibrium exists where all species have positive density, and we, thus, investigated the stability properties of this local equilibrium (Moore & de Ruiter, 2012). To the best of our knowledge, there are not sufficient data for a full species‐level parameterization of an explicit, nonlinear food web model. In practice, we could not simulate an explicit model that shows the temporal trajectory of each species population, for which there are no empirical measurements. Thus, we used random matrix theory, and for each adjacency matrix ***A*** (which describes which species interacts with which other), we generated 1000 community matrices of interaction strengths ***W***. The element ***W*** *_ij_* represents the per capita effect of species *j* on the growth rate of species *i*. In our food webs, there are three types of interactions: predator–prey (predominant), mutualism (only between plants and AMF), and competition within some of the functional groups. Predators or consumers have explicit resources in the model and, thus, can compete for resources. But competitive interactions were introduced for the basal functional groups, which have no explicit resources in the model. Also, the dynamics of organic matter in Figure 1 is not explicitly considered nor are resources for plants. Thus, species within each of the groups at the bottom of the food webs (i.e., plants, bacteria, saprotrophic fungi, and animals feeding on and likely to directly digest detritus) were allowed to compete directly for an implicit basal resource via a negative interaction term. As customary in analysis of local equilibria of food webs, we set interspecific competition coefficients lower than intraspecific coefficients (***W*** *_ji_* << −***W*** *_ii_*), a precondition of coexistence (May, 1973). For each predator–prey pair, the interaction strength of a predator (*j*) on prey (*i*) was randomly drawn from a half‐normal distribution, with mean 0 and standard deviation *σ*. Intraspecific competition (the main diagonal of ***W***) was kept constant at −1 in all simulations. We stress that we have theoretically constructed and not empirically parameterized our food webs, the only real‐world parameters being the functional topology of the web, species richness range, and some approximate information on connectance. The goal was to assess the implication of a plausible topology on the local stability property of a very large system for which it is not possible to generate a fully nonlinear representation.

### 2.3 Evaluation of stability – Step 2: matrix algebra to assess local dynamic stability

The community matrix ***W*** represents the main feature of a linearization of the system of nonlinear differential equations that describe the full dynamics of the network. We thus focused on the property of an abstract linearized system that we constructed in line with the logics of random matrix constructions (Allesina & Tang, 2012; May, 1972). In the linearized system, any perturbation (i.e., variation in species population size) *x* varies over time *t* as $x\left(t\right)=e^{\mathrm{rt}}W\cos\left(\omega t‐k\right)$, with the complex number λ=r+iω being an eigenvalue of ***W***. The perturbations are assumed to be pulse shifts in population size away from equilibrium density. The system is locally stable if the dominant eigenvalue of ***W*** has a negative real part (May, 1973; Robinson, 2004), in which case the perturbation dies out and populations return to their equilibrium value. Given the theoretical nature of our analysis, we limited ourselves to quantify stability as the inverse of the real part of the dominant eigenvalue. Given our focus on topology, we used May's definition of complexity (May, 1972) to define a number of scenarios and explore how stability was affected both by the topological properties of species richness and connectance, which we defined using real‐world information, and the quantitative property of interaction strength, which we drew from a probability distribution. Complexity $\kappa =\sigma \sqrt{\mathrm{SC}}$, where σ is the standard deviation of interaction strengths (***W*** *_ij_*), *S* is the number of species, and *C* is connectance (the proportion of interactions relative to the maximum possible). We investigated how complexity affects stability in food webs that differed in size (*S*) and variability of interaction strengths (*σ*), given the particular block structure of our food webs. We then compared the stability of our soil food webs with that of random predator–prey food webs that lack the functional structure of our plausible topology (Allesina & Tang, 2012).

We varied the value of σ from 0.1 to 3 in 6 steps. For each *σ*, we generated 1000 species‐level food webs, each one with a different number of species, connectance, and set of interaction strengths. The number of species in a group, for each food web, was randomly chosen from a uniform distribution within a range defined according to values available in the literature (Table S2). Given our general goal, we did not consider correlations between the richness of different groups, so some groups could be at their lower end of richness, whereas others could be at the upper end. To parameterize connectance, we defined a matrix of probability of interactions between any two groups that were connected in the general food web. We could only approximately guess these probabilities, based on our knowledge of the literature and consensus between the different experts in our team (see Supporting Information for more details). These probabilities define how likely it is that a connection exists between any two species belonging to the two connected groups, and they were guessed from information available in the literature (Table S3 and “Groups_Pint_REV.csv” in Appendix S1). For example, there is mixed evidence about the specificity of relationships between plants and mycorrhizal fungi (Antunes & Koyama, 2017). In uncertain cases like this, we used a probability of 0.5. It is known that arbuscular mycorrhizal fungal (AMF) hyphae are not very palatable or accessible to fungivorous microarthropods; therefore, in this and similar cases (Table S3), we used a probability of 0.2. In contrast, earthworms impact a number of groups in a very unspecific (Schwarzmüller et al., 2015), diffuse way (ingesting soil and digesting the organic fraction), and in cases such as this, we assigned a high probability of interaction (0.75). The overall connectance of the web was then an emergent property of the probability of interaction between species of any two groups. The state of the arts does not allow, to the best of our knowledge, a rigorous quantitative estimate of group–group probability of connectance. Nevertheless, we offer our team consensus to show that in principle, it is possible to model this aspect of the food web (see Supp. Info., details in Table S3). Overall, our preliminary results (not shown) were not sensitive to the particular choice we made for these parameters, which can be verified running our code (Supporting Information, R script GenerateLargeMat.R to run on Groups_Interactions_REV.csv, Groups_Pint_REV.csv and Groups_Richness_rev.csv in Appendix S1), rather they depended on the overall connectance of the food web, as expected (May, 1972).

### 2.4 The cascade model and block structure

To assess how the cascade model approximates stability, we used the approach of Allesina et al. (2015). Why the cascade model? In the cascade model, species are ordered according to a hierarchy in which a species can only consume species of lower rank. One possible mechanism behind this model is trophic interactions arranged by size, which is one of the possible factors that could create a functional hierarchy also in soil food webs (but see discussion). In Allesina et al.'s (2015) approach, the cascade model is used to decompose the interaction matrix ***W*** into a signal‐and‐noise matrix and derive an estimate of the dominant eigenvalue of ***W***. The estimate of the dominant eigenvalue of ***W*** can then be compared with the observed dominant eigenvalue (Allesina et al., 2015; Supporting Information, R code GenerateLargeMat.R in Appendix S1). The hierarchical structure of our soil food web model generates a matrix block structure. To examine more closely the role of the functional block structure of interactions on stability, we run a series of simulations in which connections between species were arranged in larger or smaller blocks of species within the interaction matrix ***W***, while keeping the connectance and other characteristics constant. In other words, the species blocks were not fully random blocks of the food webs but respected the sign structure of trophic interaction (i.e., prey–predator corresponds to a +/− interactions). We used three block sizes: 5, 15, and 25 species. For each block size, we used two values of *σ* (0.25 and 0.5), and for each combination of block size and *σ*, we generated 5000 matrices of each type: block‐structured and random (see Supporting Information for details). All matrices had 100 species and connectance 0.2. An example (with block size of 25) of MatLab script for this particular analysis is available in the Supporting Information (eigentest.m and blockmat.m in Appendix S1).

## 3 RESULTS

**FIGURE 3:**
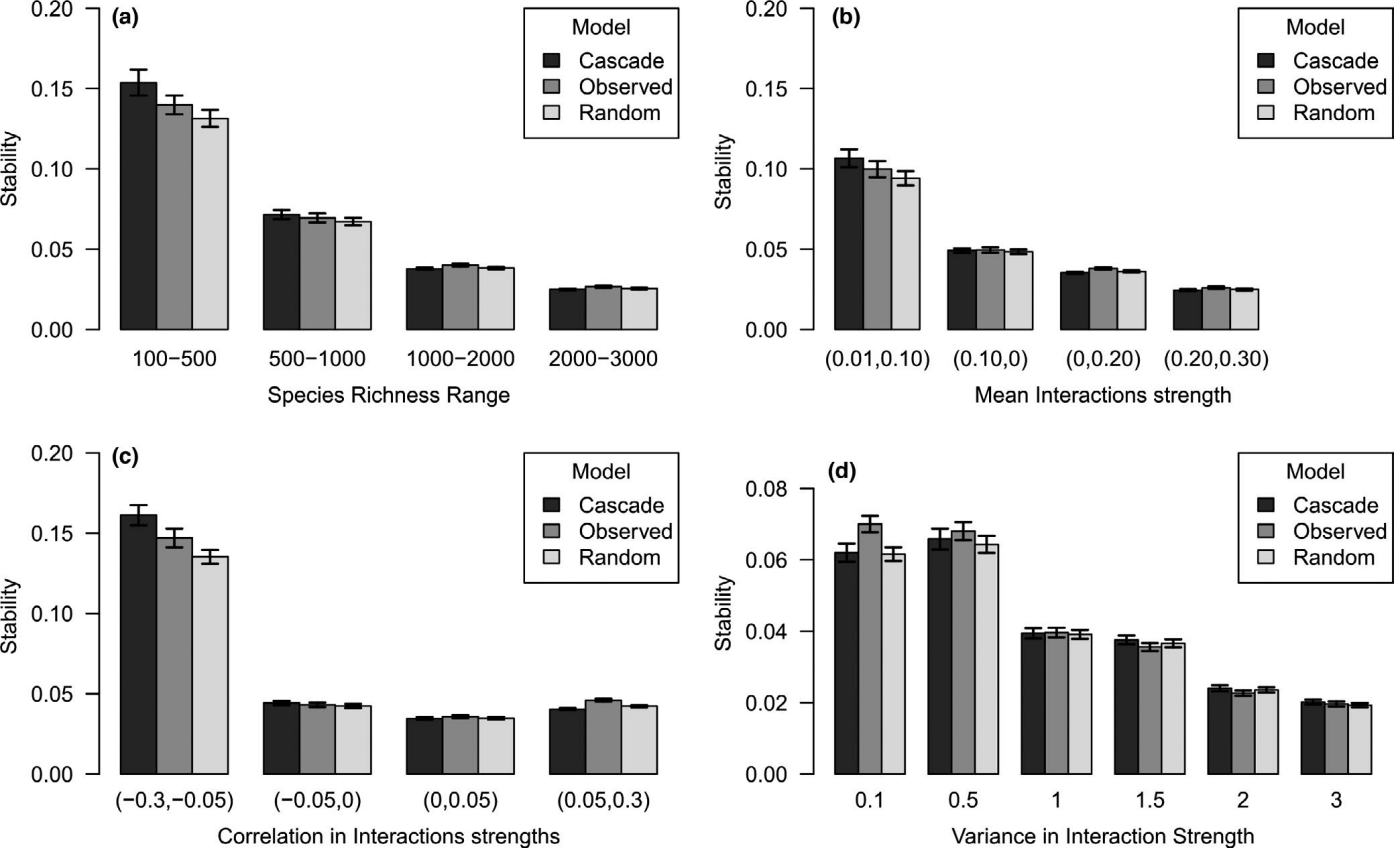
Effects of species richness (a), interaction strength (b), correlation in interaction strengths (c), and variance in interaction strength (d) on stability. Stability is measured as the inverse of the real part of the leading eigenvalue of the interaction matrix. The interaction matrix represents a linearization of the full nonlinear system around an assumed feasible equilibrium (i.e., all species at positive density). In general, an increase in complexity (i.e., an increase in either species richness, interaction strength, variance in interaction strength, or any two of, or all of these three) makes soil food webs less stable, although in various cases soil food webs would still be more stable than their random counterpart. Strongly negative and positive correlations between interaction strengths stabilize these soil food web models

**FIGURE 4:**
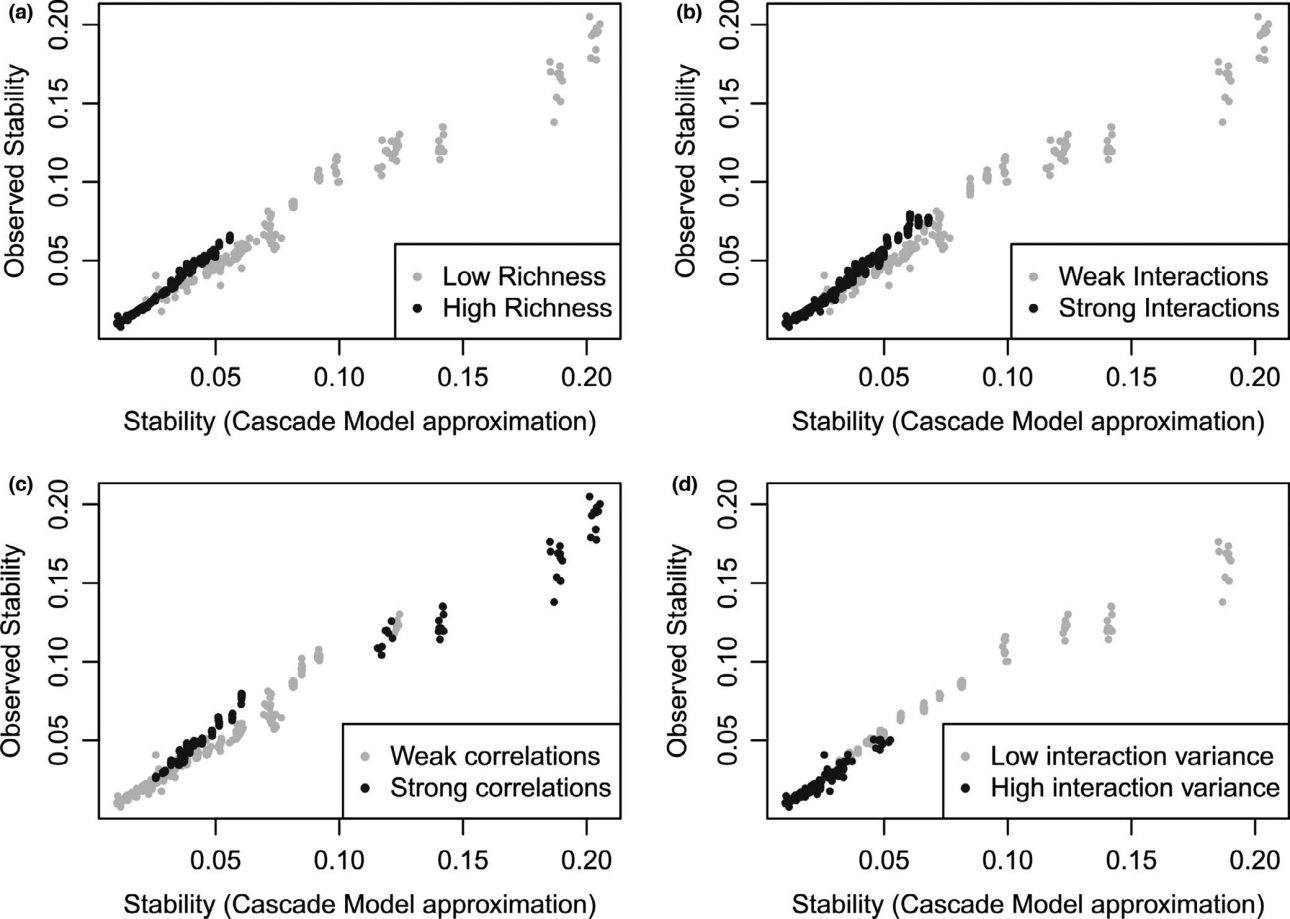
Effects of species richness (a), interaction strength (b), correlation in interaction strengths (c), and variance in interaction strength (d) on the correlation between the stability of soil food webs (*y*‐axis, observed stability) and the approximation of stability provided by the cascade model (Allesina et al., 2015). The very high correlation between the observed and approximated stability indicates the ability of the cascade model to predict the stability of soil food webs, although the precision of the prediction decreases at higher levels of stability and vary with levels of richness, interaction strength, correlation in interaction strengths, and variance in interaction strength

Increasing levels of species richness, mean interaction strength, and variance in interaction strength all tended to destabilize soil food webs (Figure 3), with patterns of variation very similar to those observed in random food webs. However, at relatively low levels of species richness (a few hundred species) and low variance in interaction strength (0.1), soil food webs were more stable than their random counterparts (i.e., all else equal but the functional structure of the network). Soil food webs also were more stable than their random counterparts at high levels of diversity (>1000 species). The approximation based on the cascade model generally overestimated the stability of soil food webs, especially at the lower levels of species richness, interaction strengths, and negative correlations between interaction strengths (Figure 3). There was, however, a strong correlation between observed (soil food web) and estimated (cascade model) stability (Figure 4), even though the approximation was less precise at high levels of stability (i.e., for smaller values of the real part of the leading eigenvalue of the matrix). Soil food webs with strong correlations between interaction strengths were more stable (Figures 3 and 4).

To further compare the stability of soil food webs with that of a random counterpart (i.e., no block structure but the same distribution of parameters), we first calculated the maximum connectance at which a random predator–prey food web would be stable, given *S* and *σ*, and then compared the obtained surface with the values of 1000 soil food web models. We observed that soil food webs can be very stable even for values of connectance, richness, and variance in interaction strength (Figure S1) that would imply instability in random predator–prey food webs (Allesina & Tang, 2012). However, depending on parameters such as correlations in interaction strengths, soil food webs may also be unstable at levels of complexity that would imply stability in random food webs with no block structure.

**FIGURE 5:**
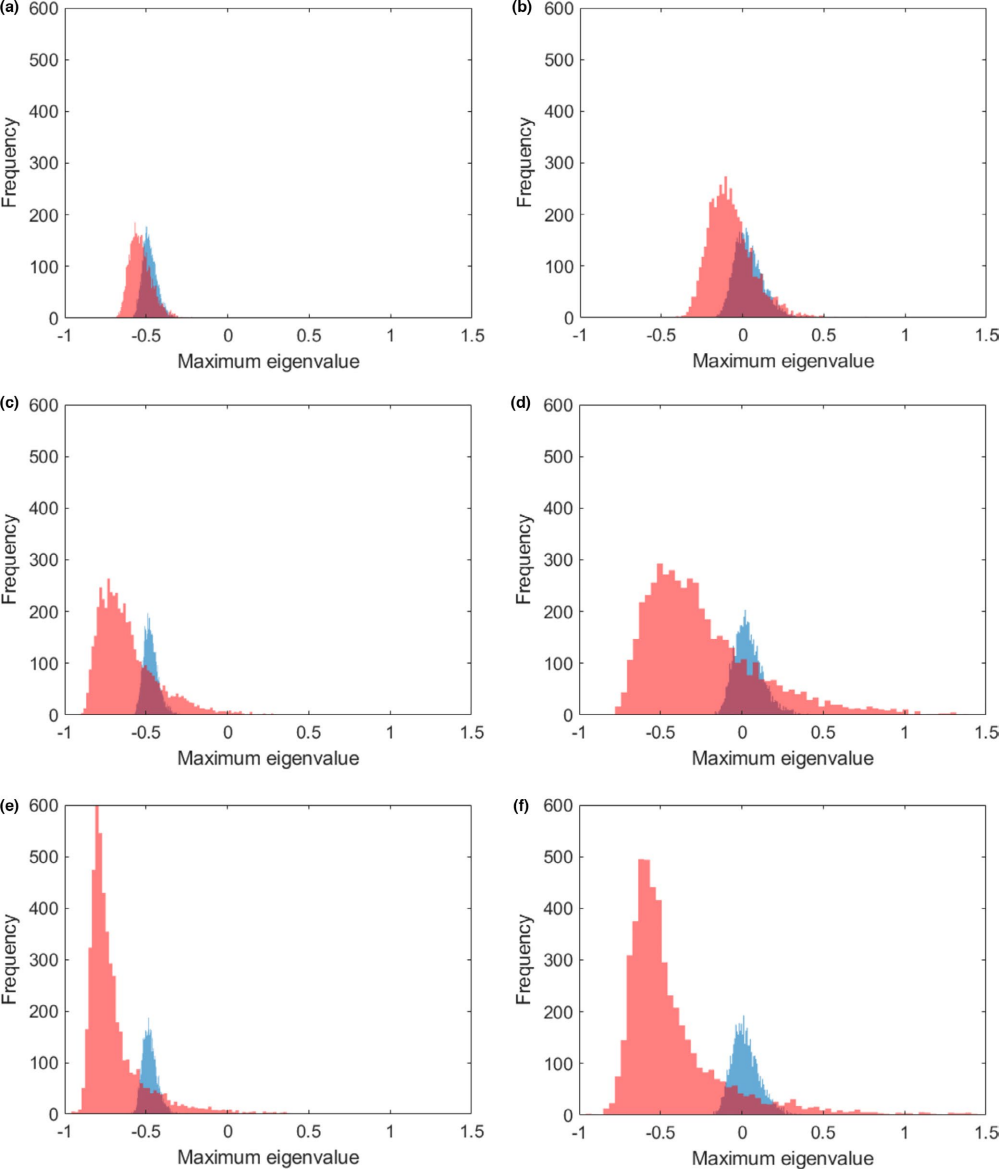
Effects of block structure within the interaction matrix and variability of interaction strengths on eigenvalue distribution. The histograms are the distribution of the maximum eigenvalue for 5000 matrices of 100 species, negative values indicating a locally stable web. Pink color is for matrices with block structure, whereas blue is for random matrices. The left column (a, c, e) is for low σ (0.25, variability of interaction strengths), and the right column (b, d, f) for high σ (0.5). The top row is for small block size (5 species), the middle row for medium block size (15 species), and the lower row for large block size (25 species). Bigger blocks implies an increased probability that the soil food web will be more stable than its fully randomized counterpart. See MatLab scripts in the Supporting Information for further details

The size of the blocks within the interactions matrix did affect the stability, everything else being equal (Figure 5). Larger blocks of species sharing connections strongly increased the stability, although more and smaller blocks decreased it. In all cases, the mean of the maximum eigenvalue for blocked matrices was more negative than that of random matrices of the same size, connectance, and variability of interaction strengths. This suggests that any internal structure in the topology of the food web will enhance stability. The eigenvalue distribution for random matrices was close to normal in all cases, whereas the distribution for blocked matrices was skewed to the right, with a long tail, particularly for larger block size and higher *σ*, which means that a small number of these food webs would be very unstable.

## 4 DISCUSSION

The main difference between random and real ecological networks is that the latter possess structure at various levels. One important level that has been analyzed extensively is the topological level (Allesina et al., 2015; Bascompte et al., 2003; Pascual & Dunne, 2006). Instead, correlation between interaction coefficients (Brose et al., 2006; Tang et al., 2014), which in nature can be caused by various factors such as body size limitations on predation, has been much less investigated. Our results show that both aspects of structure (functional block structure and correlations in interaction strengths) enhance the stability of our models of soil food webs compared with randomly assembled predator–prey networks, even when the soil food web is very large (i.e., several hundred species). This finding is particularly interesting because our theoretical models reflect the fact that real soil food webs are extremely species‐rich even at very small spatial scales (e.g., a plant root system), and our results suggest how these food webs can be complex in terms of richness and connectance and locally stable at the same time.

The classic soil food web model emerged from the hypothesis that soil food webs consist of three main energy channels, with trophic interactions arranged mostly within them, and that stability and resilience properties depended on how energy flows through those channels (de Ruiter et al., 1995; Neutel et al., 2002; Rooney & McCann, 2012; Rooney et al., 2006). New knowledge on trophic interactions in soil food webs indicates that these channels are much less distinct than previously assumed and that even at low trophic levels, many groups of soil organisms have very flexible trophic strategies (Adl & Gupta, 2006; Ballhausen & de Boer, 2016; Bradford, 2016; Chagnon et al., 2012; Geisen, 2016). This flexibility implies a high level of omnivory, blurring the classic distinction between energy channels (Bradford, 2016; de Vries & Caruso, 2016; Geisen, 2016). In addition, high species richness within functional groups that form the soil food web has not yet been included in a general analysis of the stability properties of soil ecological networks.

Our construction of soil food webs, which was based on up‐to‐date functional grouping and plausible differences in the level of species richness of different groups, identifies four new major aspects of the stability properties of soil food webs. First, relatively small but still fairly species‐rich (>300 species) soil food webs are significantly more stable than their random, predator–prey counterpart, even at very high levels of interaction strength and when connectance was at the highest possible level (i.e., high complexity). Second, the correlation between interaction strengths plays a key role in stabilizing soil food webs. One possible, although speculative, explanation is that trophic groups mostly correspond to taxonomic groups that generally differ in size (e.g., Brose et al., 2006; Wagg et al., 2014). As our analyses remain theoretical, we have no additional data to demonstrate that differences in body size between the functional groups are causally connected to the stabilizing role of correlation in interaction strengths. We just propose that a correlation between the average body size of a trophic group and the interaction strength linking that trophic group to other trophic groups can generate the type of correlation in interaction strengths that we have shown to stabilize our models (i.e., this is the formulation of an hypothesis to be tested in future experiments). Third, compared with stable random predator–prey food webs, our model of soil food webs can maintain a higher level of complexity while still retaining stability. The latter result is remarkable considering the very high species richness of soil food webs and the presence of potentially destabilizing mutualistic interactions linking plant and mycorrhizal fungi (Bever et al., 1997, 2010; Põlme et al., 2018). The diversity of soil biota is huge, especially when microbial groups are considered (Bardgett & van der Putten, 2014; Ramirez et al., 2014). Despite major difficulties in the definition of “species” at the microbial level, species richness values of soil food webs that include microbial diversity can easily be in excess of 200 or 300 species at very small spatial scales, such as the canopy of an individual herbaceous plant or its rhizosphere (Öpik et al., 2008; Wehner et al., 2014). Fourth, we have shown that the size of ‘blocks’ of species within the food web, which emerges from the presence of functional groups, influences the stability of the system: larger blocks, representing stronger aggregation of species, increased the probability of a stable result for a given level of complexity.

Our models indicate that typical species‐rich soil food webs can be locally stable at small spatial scales (e.g., the root system of a single individual plant) but that the stability of our model soil food webs would decrease with increasing spatial scale as the size of the food web would also increase in terms of number of species. In real food webs, however, the spatial distribution of species is highly structured across multiple spatial scales (Ettema & Wardle, 2002), and we speculate that a number of local, relatively isolated food webs, are likely weakly linked at larger scales to form a stable metacommunity of food webs (sensu Pillai et al., 2011). A potentially very important linkage between local soil food webs (e.g., the food web of a single rhizosphere) could be provided by the extended belowground mycorrhizal network (Barto et al., 2012). Although we did not address the implication of the metacommunity structure on soil food web stability in our models, it is well‐established that many soil taxa do possess a metacommunity spatial structure (Caruso et al., 2011; Ettema & Wardle, 2002; Lindo & Winchester, 2009), and future food web models should account for this property.

The most important limitation of our study is that our model results are purely theoretical and not based on a parameterization of actual species demography and trophic interactions. Paraphrasing May (1973), the calculation of eigenvalues of randomly constructed matrices, which represents linearized systems, is at best a caricature of the nonlinear dynamic of real soil food webs. Most importantly, the resulting local stability analysis is only one particular dimension of stability (Grimm & Wissel, 1997; Loreau & de Mazancourt, 2013) and in fact it is the only dimension that we could address with random matrix theory. Real populations can persist also out of a local equilibrium (e.g., limit cycle and chaotic attractors) and a system that is not stable in the neighbourhood of a local equilibrium might nevertheless persist when faced with perturbations over relatively long‐time scales. For example, the 24 functional groups of our food web model could each lose a high number of species in response to a perturbation, and yet, the food web could still work given that its functional structure would be preserved. The question of whether soil food webs (or indeed any food web) form an equilibrium system is also still open, although, to the best of our knowledge, a large number of soil species display a degree of persistency over time, with most sampled soil community being characterized by very high species number and with numerous species at very high density (Caruso et al., 2020). Within all these limitations, our study shows that under the assumption of equilibrium and small perturbations, theoretical soil food webs possess topological structure that allows them to be complex in terms of topology yet locally stable in terms of population dynamics. Our analysis strongly supports the general hypothesis that the stability of rich and complex food webs is driven by the correlations in interaction strength and by the block structure of the topology of the food web. Our results are thus likely to apply to any food web and imply that future theoretical and empirical analyses of these food webs should investigate the linkage between population densities, correlation between interaction strengths, and the size, and connections between functional groups of different sizes, all of which ultimately determine fluctuations in energy flow in ecosystems.

## CONFLICT OF INTEREST

The authors declare no conflict of interests.

## AUTHOR CONTRIBUTIONS

**Francisco de Castro:** Conceptualization (equal); Formal analysis (equal); Visualization (equal); Writing‐original draft (lead); Writing‐review & editing (equal). **Sina M. Adl:** Data curation (equal); Writing‐review & editing (lead). **Stefano Allesina:** Methodology (equal); Writing‐review & editing (equal). **Richard D. Bardgett:** Conceptualization (equal); Writing‐review & editing (equal). **Tom Bolger:** Writing‐review & editing (equal). **Johnathan Dalzell:** Writing‐review & editing (equal). **Mark Emmerson:** Writing‐review & editing (equal). **Thomas Fleming:** Writing‐review & editing (equal). **Diego Garlaschelli:** Conceptualization (equal); Methodology (equal); Writing‐review & editing (equal). **Jacopo Grilli:** Formal analysis (equal); Methodology (equal); Writing‐review & editing (equal). **Silja Emilia Hannula:** Conceptualization (equal); Data curation (equal); Writing‐review & editing (equal). **Franciska de Vries:** Conceptualization (equal); Data curation (equal); Methodology (equal); Writing‐review & editing (equal). **Zoë Lindo:** Data curation (equal); Methodology (equal); Writing‐review & editing (lead). **Aaron G. Maule:** Writing‐review & editing (equal). **Maarja Öpik:** Data curation (equal); Writing‐review & editing (equal). **Matthias C. Rillig:** Conceptualization (equal); Data curation (equal); Writing‐review & editing (equal). **Stavros D. Veresoglou:** Writing‐review & editing (equal). **Diana H. Wall:** Conceptualization (equal); Writing‐review & editing (equal). **Tancredi Caruso:** Conceptualization (lead); Data curation (equal); Formal analysis (equal); Funding acquisition (lead); Methodology (lead); Project administration (lead); Writing‐original draft (lead); Writing‐review & editing (lead).

## Supporting information

Supplementary Material

Appendix S1

## Data Availability

Computer codes and files to run the codes are available as Appendix S1.
