# Supplementary material for: Local stability properties of complex, species‐rich soil food webs with functional block structure
Source: Ecol Evol. 2021 Nov 3;11(22):16070–81. doi: 10.1002/ece3.8278 (PMC8601897; doi:10.1002/ece3.8278)
Supplement: Supplementary file 1 — Supplementary Material [file ECE3-11-16070-s001.docx]

**Table S1.** Functional group matrix describing the block structure of soil food webs in terms of interactions between trophic groups (See Table S3 for references). Predation (column *j* eats row *i*) is indicated by *B_ij_*= -1 and *B_ji_* = 1. All groups have self-regulatory intraspecific competition (i.e. all diagonal terms *B_ii_*= -1) but species in the first six groups (- 1 highlighted in green) also had interspecific competition within the group because they have no explicit resources in the model.

| Groups | Plant | SaproFun | Bact | DetMacroar | DetMicroar | InsLarv | PhyNem | MF | EndophFun | Enchy | RootFeedIns | InsFunPatho | FungivNem | BacterivNem | NematovFun | FungivMicroar | FungivProt | BacterivProt | PredProt | OmnNem | PredNem | PredMicroar | PredMacroar | Earthw |
| --- | --- | --- | --- | --- | --- | --- | --- | --- | --- | --- | --- | --- | --- | --- | --- | --- | --- | --- | --- | --- | --- | --- | --- | --- |
| Plant | -1 | - | - | - | - | - | -1 | 1 | -1 | - | -1 | - | - | - | - | - | - | - | - | - | - | - | - | - |
| SaproFun | - | -1 | - | - | - | - | - | - | - | - | - | - | -1 | - | - | -1 | -1 | - | - | -1 | - | - | - | -1 |
| Bact | - | - | -1 | - | - | - | - | - | - | -1 | - | - | - | -1 | - | - | - | -1 | - | -1 | - | - | - | -1 |
| DetMacroar | - | - | - | -1 | - | - | - | - | - | - | - | - | - | - | - | - | - | - | - | - | - | - | -1 | - |
| DetMicroar | - | - | - | - | -1 | - | - | - | - | - | - | - | - | - | - | - | - | - | - | - | - | -1 | -1 | - |
| InsLarv | - | - | - | - | - | -1 | - | - | - | - | - | -1 | - | - | - | - | - | - | - | - | - | - | -1 | - |
| PhyNem | 1 | - | - | - | - | - | - | - | - | - | - | - | - | - | -1 | - | - | - | - | - | -1 | -1 | - | -1 |
| MF | 1 | - | - | - | - | - | - | - | - | - | - | - | -1 | - | - | -1 | -1 | - | - | -1 | - | - | - | -1 |
| EndophFun | 1 | - | - | - | - | - | - | - | - | - | - | - | -1 | - | - | -1 | -1 | - | - | -1 | - | - | - | -1 |
| Enchy | - | - | 1 | - | - | - | - | - | - | - | - | - | - | - | - | - | - | - | - | - | - | - | -1 | - |
| RootFeedIns | 1 | - | - | - | - | - | - | - | - | - | - | -1 | - | - | - | - | - | - | - | - | - | -1 | -1 | - |
| InsFunPatho | - | - | - | - | - | 1 | - | - | - | - | 1 | - | -1 | - | - | -1 | -1 | - | - | -1 | - | - | - | -1 |
| FungivNem | - | 1 | - | - | - | - | - | 1 | 1 | - | - | 1 | - | - | -1 | - | - | - | - | - | -1 | -1 | - | -1 |
| BacterivNem | - | - | 1 | - | - | - | - | - | - | - | - | - | - | - | -1 | - | - | - | -1 | - | -1 | -1 | - | -1 |
| NematovFun | - | - | - | - | - | - | 1 | - | - | - | - | - | 1 | 1 | - | -1 | -1 | - | - | 1 | 1 | - | - | -1 |
| FungivMicroar | - | 1 | - | - | - | - | - | 1 | 1 | - | - | 1 | - | - | 1 | - | - | - | - | - | - | -1 | - | -1 |
| FungivProt | - | 1 | - | - | - | - | - | 1 | 1 | - | - | 1 | - | - | 1 | - | - | - | -1 | -1 | -1 | - | - | -1 |
| BacterivProt | - | - | 1 | - | - | - | - | - | - | - | - | - | - | - | - | - | - | - | -1 | -1 | - | - | - | -1 |
| PredProt | - | - | - | - | - | - | - | - | - | - | - | - | - | 1 | - | - | 1 | 1 | - | -1 | -1 | - | - | -1 |
| OmnNem | - | 1 | 1 | - | - | - | - | 1 | 1 | - | - | 1 | - | - | -1 | - | 1 | 1 | 1 | - | -1 | -1 | - | -1 |
| PredNem | - | - | - | - | - | - | 1 | - | - | - | - | - | 1 | 1 | -1 | - | 1 | - | 1 | 1 | - | -1 | - | -1 |
| PredMicroar | - | - | - | - | 1 | - | 1 | - | - | - | 1 | - | 1 | 1 | - | 1 | - | - | - | 1 | 1 | - | -1 | -1 |
| PredMacroar | - | - | - | 1 | 1 | 1 | - | - | - | 1 | 1 | - | - | - | - | - | - | - | - | - | - | 1 | - | - |
| Earthw | - | 1 | 1 | - | - | - | 1 | 1 | 1 | - | - | 1 | 1 | 1 | 1 | 1 | 1 | 1 | 1 | 1 | 1 | 1 | - | - |
|  |  |  |  |  |  |  |  |  |  |  |  |  |  |  |  |  |  |  |  |  |  |  |  |  |

Groups code: 1) Plant, plants; 2) SaproFun. Saprotrophic Fungi. SaproFun; 3) Bact. bacteria; 4) DetMacroar. Detritivorous macroarthropods; 5) DetMicroar. Detritivorous microarthropod; 6) InsLarv. insect larvae; 7) PhyNem. phytophagus nematodes; 8 MF mycorrhizal fungi; 9) EndophFun. endophytic fungi; 10) Enchy. enchytreids; 11) RootFeedIns. Root feeding insects; 12) InsFunPatho. fugal pathogens of insects; 13) FungivNem. fungivorous nematodes; 14) BacterivNem. bacteriovorous nematodes; 15) NematovFun. mematovorous fungi; 16) FungivMicroar. fungivorous microarthropods; 17) FungivProt. fungivorous protists; 18) BacterivProt. bacteriovorous protists; 19) PredProt. predaceous protists; 20) OmnNem. omnivorous nematodes; 21) PredNem. predaceous nematodes; 22) PredMicroar predaceous microarthropods; 23) PredMacroar. predaceous macroarthropods; 24) Earthw. earthworms

**Table S2.** Minimum and maximum species richness for each group. These values (approximated and rounded) are for all the species active in one hectare at any time, with most data coming from grasslands and woodlands. These figures are just approximation to generate random species richness values in our simulations, and have no pretence of being specific to any particular system. These values are meant to reflect the order of magnitude within which the species richness of each group could vary, with groups such as bacteria and archeas showing the highest richness, and groups such as macroarthropods the smallest values. Also, these values do not account for changes in species composition from place to place. Especially the minimum values were set to create a plausible range, given lack of information in the literature. Overall, these values represent the well known fact (see references below) that microbial communities (bacteria, fungi and protists) represent the bulk of soil biodiversity, followed by microarthropods and nematodes. These values are used as a starting point and can be changed to suit specific system when running our computer code.

| **Group (see Table 1)** | **Minimum**  **Richness** | **Maximum**  **Richness** |
| --- | --- | --- |
| Plant | 1 | 50 |
| Phytophagus nematodes | 1 | 5 |
| Mychorrizal fungi | 1 | 50 |
| Endophytic fungi | 1 | 50 |
| Saprotrophic fungi | 5 | 200 |
| Bacteria and Archaea | 50 | 2000 |
| Enchytraeids | 1 | 20 |
| Root feeding insects | 1 | 5 |
| Fungal pathogens of insects | 1 | 50 |
| Fungivorous collembolans and mites | 5 | 50 |
| Fungivorous nematodes | 5 | 10 |
| Omnivorous nematodes | 5 | 10 |
| Bacterivorous nematodes | 1 | 5 |
| Nematovorous fungi | 1 | 5 |
| Fungivorous protists | 5 | 50 |
| Bacterivorous protists | 10 | 400 |
| Predaceous protists | 5 | 50 |
| Insect larvae | 1 | 10 |
| Predaceous nematodes | 1 | 5 |
| Predaceous microarthropods | 1 | 50 |
| Predaceous macroartrhpods | 1 | 30 |
| Earthworms | 1 | 5 |
| Detritivorous macroarthrpods | 1 | 10 |
| Detritivorous microarthrpods | 1 | 10 |

**References for Table S2**

Baranová, B., Manko, P. & Jászay, T. (2014). Differences in surface-dwelling beetles of grasslands invaded and non-invaded by goldenrods (*Solidago canadensis*, *S. gigantea*) with special reference to Carabidae. *J. Insect Conserv.*, 18, 623–635.

Brown, V. & Gange, A. (1989). Herbivory by soil-dwelling insects depresses plant species richness. *Funct. Ecol.*, 667–671.

Caruso, T., Taormina, M. & Migliorini, M. (2011). Relative role of deterministic and stochastic determinants of soil animal community: a spatially explicit analysis of oribatid mites. *J. Anim. Ecol.*, 81, 214–221.

Cole, L., Bradford, M.A., Shaw, P.J. & Bardgett, R.D. (2006). The abundance, richness and functional role of soil meso-and macrofauna in temperate grassland—A case study. *Appl. Soil Ecol.*, 33, 186–198.

Dawson, L.A., Grayston, S.J., Murray, P.J. & Pratt, S.M. (2002). Root feeding behaviour of *Tipula paludosa* (Meig.)(Diptera: Tipulidae) on Lolium perenne (L.) and Trifolium repens (L.). *Soil Biol. Biochem.*, 34, 609–615.

Ekschmitt, K., Bakonyi, G., Bongers, M., Bongers, T., Boström, S., Dogan, H., *et al.* (2001). Nematode community structure as indicator of soil functioning in European grassland soils. *Eur. J. Soil Biol.*, 37, 263–268.

Fierer, N. & Jackson, R.B. (2006). The diversity and biogeography of soil bacterial communities. *Proc. Natl. Acad. Sci. U. S. A.*, 103, 626–631.

Giller, P.S. (1996). The diversity of soil communities, the “poor man”s tropical rainforest’. *Biodivers. Conserv.*, 5, 135–168.

Holmstrup, M., Damgaard, C., Schmidt, I.K., Arndal, M.F., Beier, C., Mikkelsen, T.N., *et al.* (2017). Long-term and realistic global change manipulations had low impact on diversity of soil biota in temperate heathland. *Sci. Rep.*, 7, 41388.

Hominick, W.M., Reid, A.P., Bohan, D.A. & Briscoe, B.R. (1996). Entomopathogenic nematodes: biodiversity, geographical distribution and the convention on biological diversity. *Biocontrol Sci. Technol.*, 6, 317–332.

Hunter Mark D. (2001). Out of sight, out of mind: the impacts of root‐feeding insects in natural and managed systems. *Agric. For. Entomol.*, 3, 3–9.

Lacasella, F., Gratton, C., De Felici, S., Isaia, M., Zapparoli, M., Marta, S., *et al.* (2015). Asymmetrical responses of forest and “beyond edge” arthropod communities across a forest–grassland ecotone. *Biodivers. Conserv.*, 24, 447–465.

Lindo, Z. (2014). Springtails (Hexapoda: Collembola) of the prairie grasslands of Canada. *Arthropods Can. Grassl.*, 3, 191–198.

Manu, M., Iordache, V., Băncilă, R., Bodescu, F. & Onete, M. (2016). The influence of environmental variables on soil mite communities (Acari: Mesostigmata) from overgrazed grassland ecosystems–Romania. *Ital. J. Zool.*, 83, 89–97.

Morriën, E., S.E. Hannula, B Snoek, N.R. Helmsing, H. Zweers, M. de Hollander, M-L. Bouffaud, M. Buée, W. Dimmers, H. Duyts, S. Geisen, M. Girlanda, R.I. Griffiths, H-B. Jȍrgensen, J. Jensen, P. Plassart, R.M. Schmelz, O. Schmidt, B.C. Thomson, E. Tisserant, S. Uroz, A. Winding, M. Bailey, M. Bonkowski, J. Faber, F. Martin, P. Lemanceau, W. de Boer, J.A. van Veen, and W. H. van der Putten (2017). Soil networks become more connected and take up more carbon as nature restoration progresses. Nat. Comm., 8, 14349

Öpik, M., Moora, M., Zobel, M., Saks, Ü., Wheatley, R., Wright, F., *et al.* (2008). High diversity of arbuscular mycorrhizal fungi in a boreal herb-rich coniferous forest. *New Phytol.*, 179, 867–876.

Öpik, M., Vanatoa, A., Vanatoa, E., Moora, M., Davison, J., Kalwij, J.M., *et al.* (2010). The online database MaarjAM reveals global and ecosystemic distribution patterns in arbuscular mycorrhizal fungi (Glomeromycota). *New Phytol.*, 188, 223–241.

Panesar, T.S., Marshall, V.G. & Barclay, H.J. (2001). Abundance and diversity of soil nematodes in chronosequences of coastal Douglas-fir forests on Vancouver Island, British Columbia. *Pedobiologia*, 45, 193–212.

Porras-Alfaro, A., Herrera, J., Natvig, D.O., Lipinski, K. & Sinsabaugh, R.L. (2011). Diversity and distribution of soil fungal communities in a semiarid grassland. *Mycologia*, 103, 10–21.

Rousk, J., Bååth, E., Brookes, P.C., Lauber, C.L., Lozupone, C., Caporaso, J.G., *et al.* (2010). Soil bacterial and fungal communities across a pH gradient in an arable soil. *ISME J.*, 4, 1340.

Tedersoo, L., Bahram, M., Põlme, S., Kõljalg, U., Yorou, N.S., Wijesundera, R., *et al.* (2014). Global diversity and geography of soil fungi. *science*, 346, 1256688.

Thomson, B.C., Ostle, N., McNamara, N., Bailey, M.J., Whiteley, A.S. & Griffiths, R.I. (2010). Vegetation Affects the Relative Abundances of Dominant Soil Bacterial Taxa and Soil Respiration Rates in an Upland Grassland Soil. *Microb. Ecol.*, 59, 335–343.

Thomson, B.C., Tisserant, E., Plassart, P., Uroz, S., Griffiths, R.I., Hannula, S.E., *et al.* (2015). Soil conditions and land use intensification effects on soil microbial communities across a range of European field sites. *Soil Biol. Biochem.*, 88, 403–413.

Wallinger Corinna, Staudacher Karin, Schallhart Nikolaus, Mitterrutzner Evi, Steiner Eva‐Maria, Juen Anita, *et al.* (2013). How generalist herbivores exploit belowground plant diversity in temperate grasslands. *Mol. Ecol.*, 23, 3826–3837.

Wehner, J., Powell, J.R., Muller, L.A., Caruso, T., Veresoglou, S.D., Hempel, S., *et al.* (2014). Determinants of root‐associated fungal communities within Asteraceae in a semi‐arid grassland. *J. Ecol.*, 102, 425–436.

Yanahan, A.D. & Taylor, S.J. (2014). Vegetative communities as indicators of ground beetle (Coleoptera: Carabidae) diversity. *Biodivers. Conserv.*, 23, 1591–1609.

Zimmer, M., Brauckmann, H.-J., Broll, G. & Topp, W. (2000). Correspondence analytical evaluation of factors that influence soil macro-arthropod distribution in abandoned grassland. *Pedobiologia*, 44, 695–704.

**Table S3.** A list version of functional matrix in Table S1. Group codes are as in Table S1. Prob, a qualitative estimate of probability of interactions between two groups, which we based on our interpretation of the literature cited below and consensus reached by the authors in the team, whose expertise cover all the different functional groups. Clearly, these values are offered only as a best guess from the authors team given lack of specific information in the literature. Given the lack of more specific information, we categorised probability values as high (0.75), intermediate (0.50) or low (0.2). We assigned a high probability (0.75) to pairs of groups that are very likely to interact in a relatively unspecialised way at the species level and a probability of 0.5 in uncertain cases. A relatively low (0.2) probability was assigned to cases where interactions is possible but not as frequently observed as in other high probable interactions. Note that for the low value (0.2), if two groups are connected, some interaction at the species will certainly occur but at low levels of connectance (i.e. species in one group will tend to connect only to very few species in the other group). Most importantly, preliminary analysis (now shown) indicated that the stability of the soil food web mostly depended on the overall final connectace of the food web, which is to be expected after the classical work by Robert M May (1972). The main reason why we have decided to report our team consensus guess on group to group connectance, rather than use a general randomised connectance value, is that our blocked model can in principle accommodate group-group levels of connectance, which we have also implemented in our code and can be further explored in future work.

|  | | |  |  |
| --- | --- | --- | --- | --- |
|  | Prey | Predator | | Prob |
|  | Plant | PhyNem | | 0.75 |
|  | Plant | MF | | 0.5 |
|  | Plant | EndophFun | | 0.75 |
|  | Plant | RootFeedIns | | 0.75 |
|  | PhyNem | NematovFun | | 0.2 |
|  | PhyNem | PredNem | | 0.75 |
|  | PhyNem | PredMicroar | | 0.75 |
|  | PhyNem | Earthw | | 0.75 |
|  | MF | FungivCollMite | | 0.2 |
|  | MF | FungivNem | | 0.5 |
|  | MF | OmnNem | | 0.5 |
|  | MF | FungivProt | | 0.5 |
|  | MF | Earthw | | 0.5 |
|  | EndophFun | FungivCollMite | | 0.5 |
|  | EndophFun | FungivNem | | 0.5 |
|  | EndophFun | OmnNem | | 0.5 |
|  | EndophFun | FungivProt | | 0.5 |
|  | EndophFun | Earthw | | 0.5 |
|  | SaproFun | FungivCollMite | | 0.75 |
|  | SaproFun | FungivNem | | 0.75 |
|  | SaproFun | OmnNem | | 0.5 |
|  | SaproFun | FungivProt | | 0.75 |
|  | SaproFun | Earthw | | 0.75 |
|  | Bact | Enchy | | 0.75 |
|  | Bact | OmnNem | | 0.75 |
|  | Bact | BacterivNem | | 0.75 |
|  | Bact | BacterivProt | | 0.75 |
|  | Bact | Earthw | | 0.75 |
|  | Enchy | PredMacroar | | 0.5 |
|  | RootFeedIns | InsFunPatho | | 0.2 |
|  | RootFeedIns | PredMacroar | | 0.5 |
|  | InsFunPatho | FungivCollMite | | 0.75 |
|  | InsFunPatho | FungivNem | | 0.75 |
|  | InsFunPatho | OmnNem | | 0.75 |
|  | InsFunPatho | FungivProt | | 0.75 |
|  | InsFunPatho | Earthw | | 0.75 |
|  | FungivCollMite | PredMicroar | | 0.75 |
|  | FungivCollMite | PredMacroar | | 0.75 |
|  | FungivCollMite | Earthw | | 0.75 |
|  | FungivNem | NematovFun | | 0.75 |
|  | FungivNem | PredNem | | 0.75 |
|  | FungivNem | PredMicroar | | 0.75 |
|  | FungivNem | Earthw | | 0.75 |
|  | OmnNem | NematovFun | | 0.5 |
|  | OmnNem | PredNem | | 0.5 |
|  | OmnNem | PredMicroar | | 0.5 |
|  | OmnNem | Earthw | | 0.5 |
|  | BacterivNem | NematovFun | | 0.75 |
|  | BacterivNem | PredNem | | 0.75 |
|  | BacterivNem | PredMicroar | | 0.75 |
|  | BacterivNem | Earthw | | 0.75 |
|  | NematovFun | FungivCollMite | | 0.2 |
|  | NematovFun | FungivProt | | 0.75 |
|  | NematovFun | Earthw | | 0.75 |
|  | FungivProt | OmnNem | | 0.75 |
|  | FungivProt | PredProt | | 0.75 |
|  | FungivProt | PredNem | | 0.75 |
|  | FungivProt | Earthw | | 0.2 |
|  | BacterivProt | OmnNem | | 0.75 |
|  | BacterivProt | PredProt | | 0.75 |
|  | BacterivProt | Earthw | | 0.75 |
|  | PredProt | OmnNem | | 0.5 |
|  | PredProt | Earthw | | 0.75 |
|  | InsLarv | InsFunPatho | | 0.2 |
|  | InsLarv | PredMacroar | | 0.75 |
|  | PredNem | NematovFun | | 0.5 |
|  | PredNem | PredMicroar | | 0.75 |
|  | PredNem | Earthw | | 0.75 |
|  | PredMicroar | PredMacroar | | 0.75 |
|  | PredMicroar | Earthw | | 0.75 |
|  | DetMacroar | PredMacroar | | 0.2 |
|  | DetMicroar | PredMicroar | | 0.75 |

**References for Table S3**

Averill, C. (2016). Slowed decomposition in ectomycorrhizal ecosystems is independent of plant chemistry. *Soil Biol. Biochem.*, 102, 52–54.

Ballhausen, M.-B. & de Boer, W. (2016). The sapro-rhizosphere: Carbon flow from saprotrophic fungi into fungus-feeding bacteria. *Soil Biol. Biochem.*, 102, 14–17.

Bever, J.D., Dickie, I.A., Facelli, E., Facelli, J.M., Klironomos, J., Moora, M., *et al.* (2010). Rooting theories of plant community ecology in microbial interactions. *Trends Ecol. Evol. Pers. Ed.*, 25, 468–478.

Bever, J.D., Westover, K.M. & Antonovics, J. (1997). Incorporating the soil community into plant population dynamics: The utility of the feedback approach. *J. Ecol.*, 85, 561–573.

Crotty, F.V., Adl, S.M., Blackshaw, R., Inger, R., & Murray, P. (2014). Divergence of feeding channels within the soil food web determined by ecosystem type. Ecol and Evol, 4(1) 1-13.

Crotty, F.V. Adl, S.M., Blackshaw, R.P., & Murray J.P. 2012. Using stable isotopes to differentiate trophic feeding channels within soil food webs. J Eukaryotic Microbiol 59(6), 520-526.

Crotty, F.V., Adl, S.M., Blackshaw, R.P., & Murray J.P. 2013. Measuring soil protist respiration and ingestion rates using stable isotopes. Soil Biol Biochem 57, 919-921.

Crowther, T.W. & A’Bear, A.D. (2012). Impacts of grazing soil fauna on decomposer fungi are species-specific and density-dependent. *Fungal Ecol.*, 5, 277–281.

Crowther, T.W., Boddy, L. & Jones, T.H. (2012). Functional and ecological consequences of saprotrophic fungus–grazer interactions. *ISME J.*, 6, 1992.

Davison, J., Moora, M., Öpik, M., Adholeya, A., Ainsaar, L., Bâ, A., *et al.* (2015). Global assessment of arbuscular mycorrhizal fungus diversity reveals very low endemism. *Science*, 349, 970.

Duhamel, M., Pel, R., Ooms, A., Bücking, H., Jansa, J., Ellers, J., *et al.* (2013). Do fungivores trigger the transfer of protective metabolites from host plants to arbuscular mycorrhizal hyphae? *Ecology*, 94, 2019–2029.

Ferris, H. (2010). Form and function: Metabolic footprints of nematodes in the soil food web. *Eur. J. Soil Biol.*, 46, 97–104.

Geisen, S., Mitchell, E.A., Wilkinson, D.M., Adl, S., Bonkowski, M., Brown, M.W., *et al.* (2017). Soil protistology rebooted: 30 fundamental questions to start with. *Soil Biol. Biochem.*, 111, 94–103.

King, J.R. (2016). Where do eusocial insects fit into soil food webs? *Soil Biol. Biochem.*, 102, 55–62.

Klironomos, J., Bednarczuk, E. & Neville, J. (1999). Reproductive significance of feeding on saprobic and arbuscular mycorrhizal fungi by the collembolan, Folsomia candida. *Funct. Ecol.*, 13, 756–761.

Leff, J.W., Bardgett, R.D., Wilkinson, A., Jackson, B.G., Pritchard, W.J., De Long, J.R., *et al.* (2018). Predicting the structure of soil communities from plant community taxonomy, phylogeny, and traits. *ISME J.*

López, M.G., Lidón, J.J., Aza, D.T. & Cosín, D.D. (2006). Is *Hormogaster elisae* (Oligochaeta, Hormogastridae) a predator of mites and springtails? *Eur. J. Soil Biol.*, 42, S186–S190.

Moore, J.C., Walter, D.E. & Hunt, H.W. (1988). Arthropod regulation of micro-and mesobiota in below-ground detrital food webs. *Annu. Rev. Entomol.*, 33, 419–435.

Ngosong, C., Raupp, J., Scheu, S. & Ruess, L. (2009). Low importance for a fungal based food web in arable soils under mineral and organic fertilization indicated by Collembola grazers. *Soil Biol. Biochem.*, 41, 2308–2317.

Soong, J.L. & Nielsen, U.N. (2016). The role of microarthropods in emerging models of soil organic matter. *Soil Biol. Biochem.*, 102, 37–39.

Treseder, K.K. & Lennon, J.T. (2015). Fungal traits that drive ecosystem dynamics on land. *Microbiol. Mol. Biol. Rev.*, 79, 243–262.

Van Der Heijden, M.G.A., Bardgett, R.D. & Van Straalen, N.M. (2008). The unseen majority: soil microbes as drivers of plant diversity and productivity in terrestrial ecosystems. *Ecol. Lett.*, 11, 296–310.

de Vries, F.T. & Caruso, T. (2016). Eating from the same plate? Revisiting the role of labile carbon inputs in the soil food web. *Soil Biol. Biochem.*, 102, 4–9.

Walter, D.E. & Ikonen, E.K. (1989). Species, guilds, and functional groups: taxonomy and behavior in nematophagous arthropods. *J. Nematol.*, 21, 315.

Wolkovich, E. (2016). Reticulated channels in soil food webs. *Soil Biol. Biochem.*, 102, 18–21.

Yeates, G.W., Bongers, T. d, De Goede, R., Freckman, D. & Georgieva, S. (1993). Feeding habits in soil nematode families and genera—an outline for soil ecologists. *J. Nematol.*, 25, 315.

Figure S1


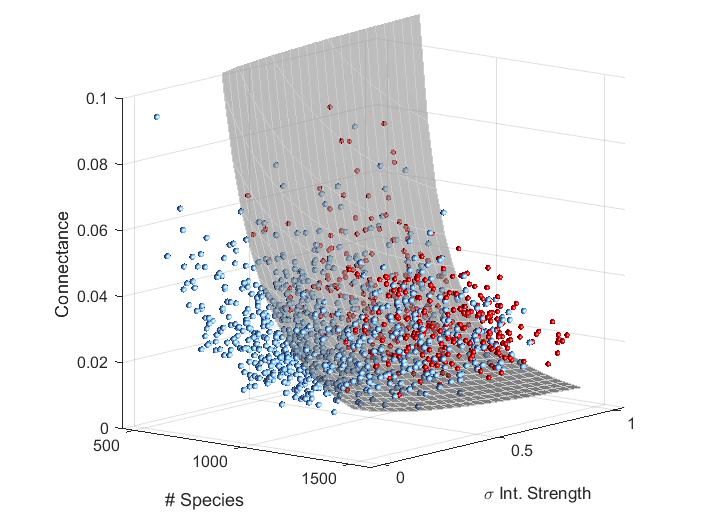


Stability of soil food webs of variable size (i.e., species richness S), variance σ in interaction strengths and connectance. Each dot represents one food web: blue food webs are stable, red are unstable. The grey surface represents the maximum connectance for a random predator-prey food web to be stable given S and variance (Allesina & Tang 2014). A large fraction of stable soil food webs can be more densely connected than random predator-prey food webs but some soil food webs can also be unstable.
